# Minimally Oxidized-LDL-Driven Alterations in the Level of Pathological Mediators and Biological Processes in Carotid Atherosclerosis

**DOI:** 10.26502/fccm.92920251

**Published:** 2022-04-12

**Authors:** Finosh G Thankam, Taj Rai, Jeffrey Liu, Jonathan Tam, Devendra K Agrawal

**Affiliations:** Department of Translational Research, College of Osteopathic Medicine of the Pacific, Western University of Health Sciences, Pomona, California, 91766, USA

**Keywords:** Atherosclerosis, Carotid stenosis, Cerebral infarction, Cerebrovascular disease, Inflammation, Oxidized LDL

## Abstract

The global burden of cerebrovascular disease, especially cerebral infarction has been increasing at an alarming rate with the atherosclerosis in carotid arteries as the primary risk factor. Despite the active involvement of minimally oxidized LDL (oxLDL) in atherosclerosis, limited information is available regarding the role of oxLDL in the pathogenesis of cerebrovascular diseases. The present study utilized the carotid bifurcation tissues and isolated carotid SMCs challenged with oxLDL from clinically relevant minimally invasive minimally-oxLDL-induced carotid atheroma microswine model to examine the levels of pro-atherogenic and pro-inflammatory mediators and cellular processes following immunostaining approaches. The immunopositivity of IL18, PDGFRA, IL17, LOX1, TLR4, MYF5, IL1B, and PDPN were increased in the carotid artery bifurcation tissues with a concomitant decrease in DAMPs, HMGB1 and S100B in oxLDL (600μg)-treated group compared to non-intervention control. Moreover, the cultured SMCs displayed increased level of IL18, LOX1, TLR4, MYF5, NLRP3, and PDPN upon challenging with oxLDL (100 mg/ml) compared to non-treatment control. In addition, the SMCs treated with oxLDL were resistant to the peroxidation of lipids as evident from lipid peroxidation staining. Also, the oxLDL displayed compromised mitochondrial membrane potential based on mitochondrial pore transition assay and increased hypertrophy due to decreased level of microtubules. Overall, oxLDL alters the expression status of pathological mediators and multiple biological processes in carotid SMCs aggravating carotid atherosclerosis. The understanding regarding the molecular mechanisms underlying oxLDL-driven pathological events would open novel translational avenues in the management of carotid atherosclerosis.

## 1. Introduction

Ischemic stroke remains the most morbid and mortal cerebrovascular disease accounting for ~5 million death worldwide, and the global burden of cerebral infarction has been increasing at an alarming rate [1, 2]. Instability of atherosclerotic plaque due to hyperlipidemia in the carotid arteries is the primary risk factor leading to ischemic stroke [3]. The extent of atherosclerotic lesions at the common carotid, internal carotid branch and/or carotid bifurcation determines the degree of cerebrovascular pathology [3]. Minimally oxLDL is recognized by LDL receptors (LDLR) but not by most of the scavenging receptors (SR) whereas fully oxLDL is recognized by SR but not by native LDLR. Also, minimally oxLDL is more potent in pro-atheromatous reactions as it primes the foam cell formation than the fully oxLDL [4, 5]. In addition, the oxidative modification of native LDL and the downstream pathological signaling of oxLDL through the activation of lectin-like oxLDL receptor (LOX-1) in the endothelial cells and smooth muscle cells (SMCs) induce a cascade of pro-inflammatory episodes promoting leukocyte chemotaxis, platelet aggregation, foam cell formation and subsequently contributing to plaque formation. [1, 6] However, limited information is available in the active involvement of oxLDL in the pathogenesis of cerebrovascular diseases and the prognosis of ischemic stroke [1].

The active involvement of damage associated molecular patterns (DAMPs) has been established in atherosclerosis and the major DAMP molecules including high mobility group box 1 (HMGB1) and S100B are crucial in priming the initial pathological events in atherosclerosis thereby sustaining the inflammatory signals [7, 8, 9]. The sterile inflammatory events elicited by the DAMPs in the presence of oxLDL sustain the upregulation of pro-inflammatory cytokines including IL-17, IL-18 and IL-1β mainly via TLR4 signaling [10, 11, 12]. Additionally, podoplanin (PDPN) functioning as an endogenous ligand for C-type lectin-like receptor 2 (CLEC-2) has been identified as a contributor for advanced stage atherosclerosis [13]. Similarly, Myf5 (identified to be the marker for adipocyte progenitor/precursor cells) expression has been associated with atherosclerosis in SMC proliferation to initiate neointimal hyperplasia (NIH) [14, 15]. PDGFRα is another key mediator in activating SMCs [16]. Furthermore, increased level of oxLDL significantly alters multiple biological processes compromising the integrity of SMCs. However, limited information is available regarding the involvement of these mediators in cerebrovascular atherosclerosis and further understanding of their association in carotid atheroma formation is promising for developing novel pharmacological approaches. Therefore, we aimed to investigate the influence of oxLDL in the expression status of these mediators and the alterations in the biological status of SMCs. The present study utilized the carotid bifurcation tissues and isolated carotid SMCs from clinically relevant minimally invasive minimally-oxLDL-induced carotid atheroma microswine model applying immunostaining approaches.

## 2. Material and Methods

### 2.1 Animal model

The Institutional Animal Care and Use Committee of Creighton University, Omaha, Nebraska, USA approved the animal procedures (Protocol #1017). The *in vivo* procedures and collection of tissues reported in this study were performed at Creighton University, but all experiments in the harvested tissues were conducted at the current institution. Female Yucatan microswine of 4–6 months old weighing ~25kg (Sinclair Bio-resources, MO, USA) were recruited in the studies. Three microswine were maintained in 12-hour light/dark cycle and fed with a high calorie atherogenic diet (Research diet Inc. USA) consisting of (51% carbohydrate, 20% protein, 10% fat with 4% cholesterol). Atheroma at the junction of the common and internal carotid arteries were induced by replenishing minimally oxLDL in the vicinity of the endothelial damage created with balloon angioplasty using an appropriately sized catheter.

The minimally ox-LDL was prepared from swine blood LDL using controlled oxidation following the previous protocol [17]. Potassium bromide (KBr) was used to adjust the density of microswine plasma to 1.063 g/mL using the formula [plasma (mL) × 0.0834 = KBr (g)] and LDL was isolated by ultra-centrifugation at 100,000g for 24 h. The LDL was purified by dialysis and oxidized using 5μM CuSO_4_ for two hours which was further purified by dialysis to form minimally ox-LDL [18]. The level of oxidation was quantified by TBARS concentration using commercially available ELISA kit (Cayman Chemical, Cat#: 10009055) following the manufacturer’s instructions. The 600μg minimally oxLDL in PBS was injected (one-time injection) at the site of balloon angioplasty-induced injury using an angiography catheter. Endothelial damage was created by scratching the intimal layer with inflated balloon followed by placing the balloon distally to the site of injury to block the blood flow prior to the injection. The blockage of blood flow was continued for ~5 min post-injection for ensuring the bioavailability of minimally oxLDL in the compromised endothelium.

The animals were maintained under high calorie diet for 6 months, sacrificed using euthanasia solution (mixture of 0.78 mg/Kg pentobarbital and 0.1 mg/Kg phenytoin sodium, IV; death was confirmed by the absence of heartbeat and respiration for 5 min) and the carotid junctions were harvested for further studies. Additionally, the criteria for early euthanasia were set for the animal that failed to respond to treatment, remains in poor general condition and/or in discomfort. Vocalization, hunched posture, hypothermia, wound dehiscence, immobility, inability to eat, self-mutilation, >20% weight loss and/or lack of response to the remedial treatments were closely monitored for early euthanasia. No mortality or adversities were observed during the study. Uninjured carotid junction tissues from the contralateral side without any treatment served as control.

### 2.2 Histology

The harvested vessels were formalin-fixed and processed for standard paraffin embedding for histomorphometry. The deparaffinized sections of 5μm thickness were used for H&E and trichrome staining for assessing the vascular morphology and ECM organization following our previous protocols [19, 20]. The stained slides were mounted with xylene-based mounting media and imaged using a slide scanner microscope attached with an imaging camera (Leica Thunder, Germany).

### 2.3 Immunofluorescence

The tissue sections were analyzed for the expression of protein biomarkers using immunostaining following our previous protocol [21]. Primary antibodies (1:300 dilution) against IL18 (ab106939), S100B (ab52642), PDGFRα (ab124392), HMGB1 (ab11354), IL17 (A20200617607), LOX (ab60178), TLR4 (ab13556), Myf5 (ab125301), IL-1β (ab156791), and podoplanin (ab10274) were used for immunostaining and corresponding fluorochrome conjugated secondary antibodies with a dilution of 1:400 was used for the detection. Nuclei were counterstained with 4′,6-diamidino-2-phenylindole (DAPI) and imaged using a fluorescent slide scanner microscope (Leica Thunder, Germany). A negative control (with secondary antibodies alone), treated in a similar manner, was used to fix the exposure time and to minimize the background. The images were analyzed with ImageJ software to quantify the mean fluorescence intensity (MFI), normalizing with the number of nuclei to calculate the variation with respect to control (VRC) and the results were expressed as log_2_ fold-change based on VRC. Average MFI calculated from 3–4 images acquired randomly from each specimen was used for VRC calculation and statistical analysis [22].

### 2.4 SMC isolation, culture, treatment, and immunostaining

SMCs were isolated from the common carotid arteries using collagenase digestion method, maintained in standard culture conditions and characterized following our previous report [23]. The cells were cultured in 8-well chamber slides and treated with LDL (200mg/ml) and oxLDL (100mg/ml) overnight, fixed with formalin, and immunostaining was performed following the above-mentioned protocol. The proteins of interest were IL18, S100B, PDGFRα, HMGB1, LOX1, TLR4, Myf5, IL-1β, podoplanin, αSMA, and NLRP3 (ab214185).

### 2.5 Lipid Peroxidation assay

Lipid peroxidation was assessed using Image-iT Lipid Peroxidation kit (C10445, Life technologies) following the manufacturer’s instructions. Briefly, the SMCs grown in chamber slides, treated with LDL and oxLDL overnight, washed with serum-free DMEM, and incubated in 200μl of Image-iT® Lipid Peroxidation Sensor (Component A) (10mM in DMSO) at 37°C for 30 minutes. After incubation, the cells were washed with serum-free DMEM and immediately imaged under fluorescence slide scanner (VS120-S6-W, Olympus) at 590 nm and 510 nm. The cells treated with LPS (100μM) (E. coli O111:B4) (Cat# L3024, Sigma Aldrich) were used as treatment control and the untreated SMCs were used as controls. The extent of lipid peroxidation was given by the ratio of the emission fluorescence intensities at 590nm to 510nm. SMCs treated with cumene hydroperoxide (CuP) (Component B) at a final concentration of 100μM for 2 hours at 37°C in each group served as the positive control.

### 2.6 Mitochondrial pore transition assay

The mitochondrial membrane/pore integrity was assessed using Image-iT LIVE mitochondrial transition pore assay Kit (I35103, Invitrogen) following our previous report [24]. Briefly, the SMCs were grown in chamber slides, treated with LDL and oxLDL overnight and were washed with serum free DMEM and incubated in 200μl labeling solution (1mM calcein AM, 200μM MitoTracker Red, 1mM Hoechst 33342, and 1M CoCl_2_) at 37°C for 15 min. After incubation, the cells were washed with serum free DMEM, mounted using serum free DMEM and live cell imaging was performed using a fluorescence slide scanner (Leica Thunder). The untreated SMCs and LPS (100μM)-treated SMCs were used as control. The experiments were run in triplicate, the MFI values were normalized to 100 cells and were expressed as variation with respect to control.

### 2.7 Microtubule staining and cell spreading assay

The microtubule integrity was assessed using ViaFluor 488 Live Cell Microtubule Stain kit (70062-T, bioitum) following the manufacturer’s instructions. Briefly, the SMCs were treated with LDL and oxLDL overnight, washed with serum-free DMEM and incubated in 200μl probe-containing medium (1μl/ml) at 37°C for 30 minutes. After incubation, the cells were washed with serum-free DMEM and immediately imaged under fluorescence slide scanner (Leica Thunder). The untreated SMCs and LPS (100μM)-treated SMCs were used as control and the experiments were run in triplicate. The MFI values were quantified using ImageJ program, normalized with the number of nuclei, and were expressed as Log_2_ Fold-Change (FC) with respect to control. Individual cells from the images from each group were randomly assessed for the area (n=22) and perimeter (n=20) to examine the cell spreading and the results were expressed as Log_2_ FC.

### 2.8 Statistical analysis

The results from swine (n=3) tissues and cell culture experiments (n=3) were expressed as mean ± SEM. The MFI and nuclei counts were calculated using ImageJ software following our previous report [23]. The mean MFI was determined by averaging the MFI of 2–5 images randomly acquired from different fields of the tissue specimen and the cultured SMCs. The statistical significance for the *in vivo* experiments was calculated by Mann Whitney test employing GraphPad Prism software. The out-layer images/specimen having background fluorescence were omitted from the analysis. For in vitro experiments one-way ANOVA with two-stage linear step-up procedure of Benjamini, Krieger and Yekutieli test was employed. The level of significance was set at *P<0.05* for all experiments. The *p<0.05* values were considered significant.

## 3. Results

Carotid atheroma was successfully established in Yucatan microswine following catheter-based minimally invasive endothelial injury and subsequent local administration of oxLDL simulating native atheroma formation. The animals were given proper care and no mortality was observed during the experiments. The level of LDL oxidation was determined by TBARS assay following 2h oxidation using CuSO4 (5μM) which displayed 2.42 μM MDA (malonedialdehyde) which confirms that the preparation was minimally-oxidized LDL as reported elsewhere [4].

### 3.1 Histology

**Figure 1:**
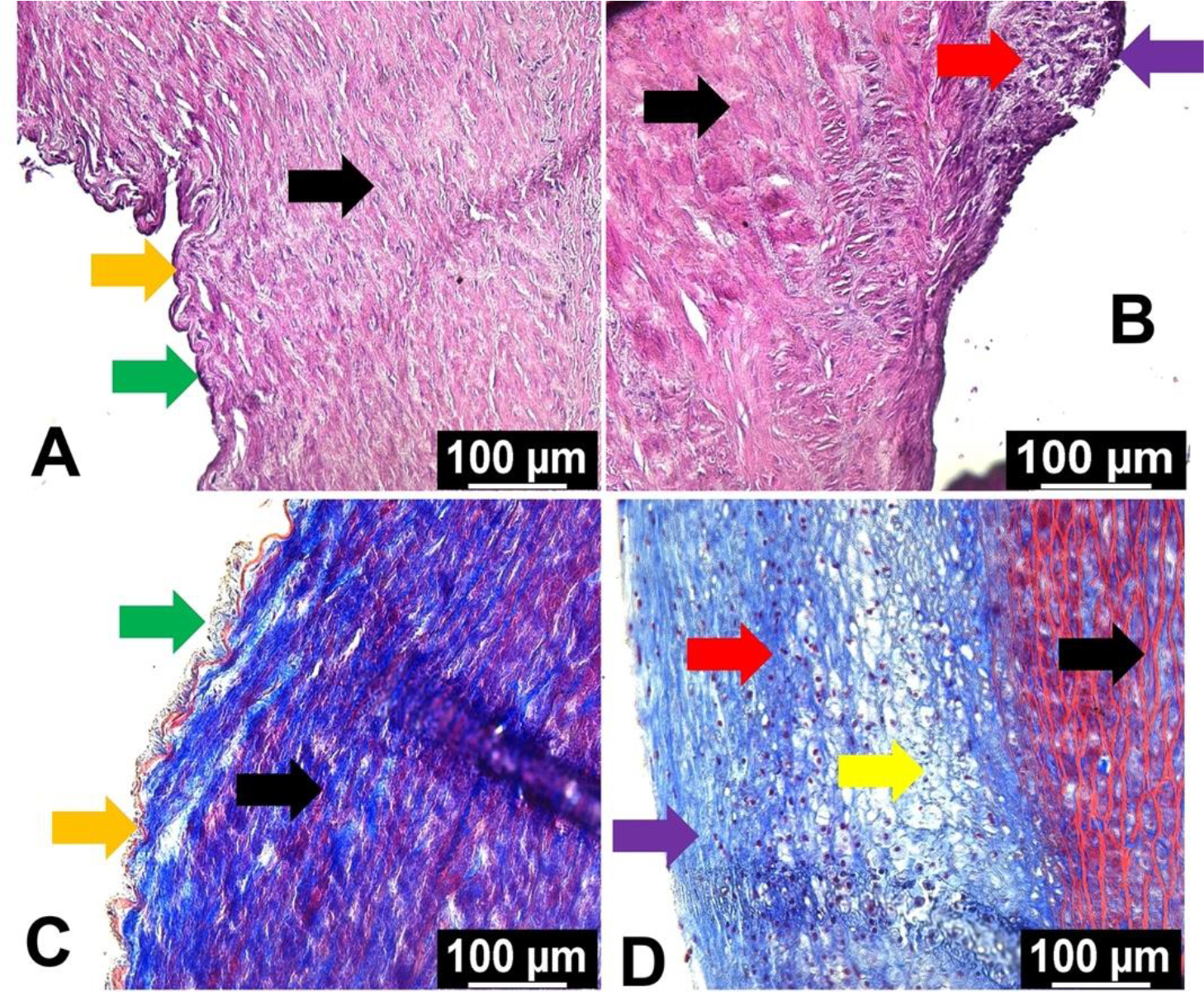
Histological examination by H&E and trichrome staining: (A) and (B) represent H&E staining of the carotid arteries of control and oxLDL treatment groups and (C) and (D) represent trichrome staining of the carotid arteries of control and oxLDL treatment groups, respectively. Black arrows point the medial layer, green arrows represent endothelial layer, orange arrows indicate internal elastic membrane, red arrows highlight plaque formation, violet arrows show fibrous cap, and yellow arrow displays the lipid rich necrotic core and foam cell formation.

The histological examinations using H&E and trichrome staining revealed considerable alterations in the morphometry of carotid arteries following oxLDL treatment compared to non-treatment control (Figures 1A–1D). In both the H&E and trichrome staining, the medial layer (black arrows) was disorganized in the oxLDL group whereas the control group displayed normal histology (Figures 1B and 1D). Additionally, the control group displayed intact endothelium and internal elastic membrane as pointed with green and orange arrows, respectively (Figures 1A and 1C).

The oxLDL treatment group displayed plaque formation (red arrows) with intact fibrous cap (violet arrows) as evident in both H&E and trichrome staining (Figures 1B and 1D). Additionally, lipid-rich necrotic core and foam cell formation were evident in the oxLDL treatment group as indicated by the yellow arrow (Figures 1B and 1D). Overall, H&E and trichrome staining demonstrated that the oxLDL treatment accelerated neointimal hyperplasia with necrotic core and matured plaque formation.

### 3.2 Immunofluorescence

**Figure 2:**
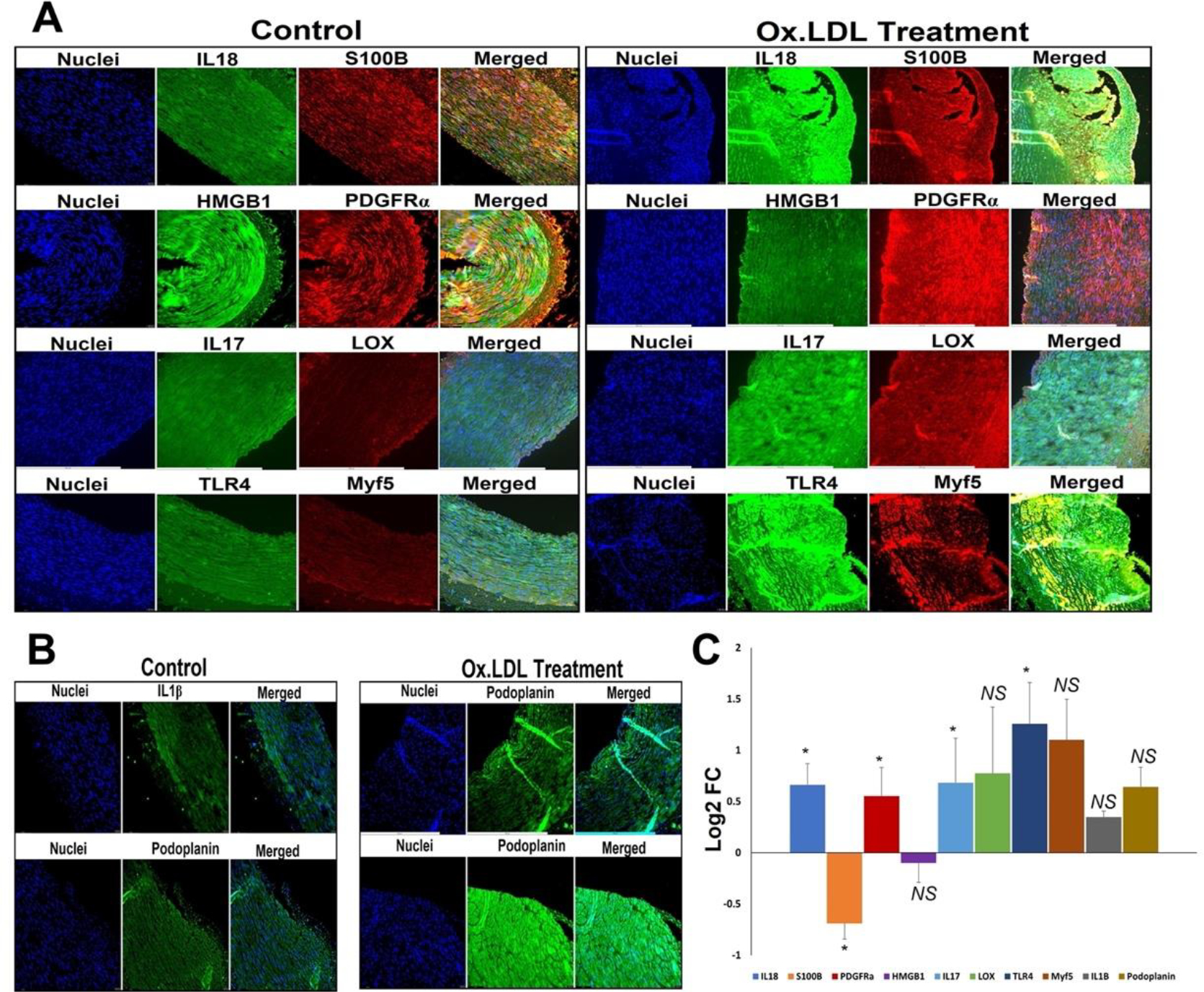
Representative images for the (A) immuno-double staining on the carotid vessels for the expression of IL18 and S100B, PDGFRα and HMGB1, IL17 and LOX, and TLR4, and Myf5 showing the altered expression in the oxLDL treatment group vessels, and (B) immunostaining on the carotid vessels for the expression of IL1β, and podoplanin showing the altered expression in the oxLDL treatment group vessels. Images in the left panel are histological sections of control vessel and at the right panel represent oxLDL treatment group. Images in the left column of each panel show nuclear staining with DAPI; the images in the middle column/s show expression of proteins while the images in the right column show overlay staining with DAPI. Images were acquired at 20x magnification. (C) The quantification of gene expression expressed as Log_2_ FC where the graph represents mean values with standard error. The statistical significance based on Mann Whitney test is represented in the figure (* *P<0.05 and NS - non-significant*).

The protein expression of proinflammatory cytokine IL-18 was significantly increased in the oxLDL treatment group compared to the control (Figures 2A and 2C). The protein expression of DAMPs S100B and HMGB1 was decreased in oxLDL treatment group compared to the control; however, the decrease in HMGB1 was statistically not significant (Figures 2A and 2C). The protein expression of PDGFRα, IL-17 (P=0.05) and TLR4 was significantly increased in the oxLDL treatment group compared to the control; however, the increase in LOX, Myf5, IL-1β, and podoplanin (P=0.10) was statistically not significant (Figures 2A, 2B and 2C).

**Figure 3:**
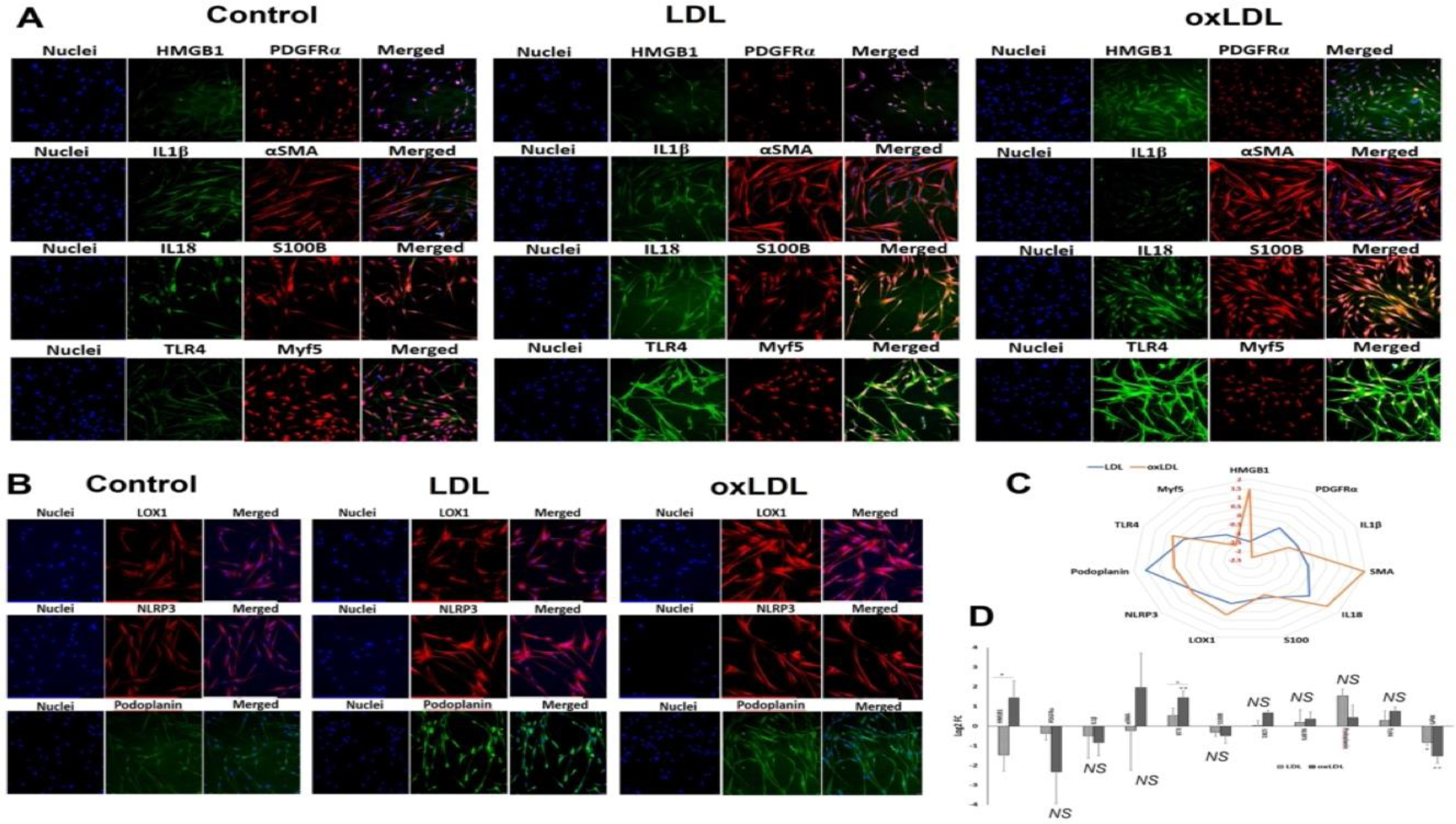
Representative images for the (A) immuno-double staining on the carotid SMCs for the expression of IL18 and S100B, PDGFRα and HMGB1, αSMA and IL1β, and TLR4, and Myf5 showing the altered expression in the LDL and oxLDL treatment group vessels, and (B) immunostaining on the carotid vessels for the expression of LOX1, NLRP3, and podoplanin showing the altered expression in the LD and oxLDL treatment group vessels. Images in the left panel represent the control group, middle panel represent the LDL treatment group and at the right panel represent oxLDL treatment group. Images in the left column of each panel show nuclear staining with DAPI; the images in the middle column/s show expression of proteins while the images in the right column show overlay staining with DAPI. Images were acquired at 20x magnification. (C) Radar plot showing the extent of protein expression mostly favoring oxLDL treatment. (D) The quantification of gene expression expressed as Log_2_ FC where the graph represents mean values with standard error. The statistical significance based on one-way ANOVA test is represented in the figure (*line indicates comparison between LDL and oxLDL groups,* * *P<0.05,* ** *P<0.01, and NS - non-significant*).

The SMCs were isolated from carotid artery, treated with LDL and oxLDL and the expression of protein mediators was examined. The protein expression of HMGB1 was significantly increased in oxLDL treatment when compared to the LDL group where the LDL group displayed decreased expression compared to control; however, the decrease was statistically not significant (Figures 3A and 3D). The expression of PDGFRα was decreased in LDL and oxLDL groups where the decrease was prominent in the oxLDL treatment; however, the decrease was statistically not significant in both the groups (Figures 3A and 3D). Similar trend was exhibited by IL1β expression (Figures 3A and 3D). The protein level of αSMA was increased in oxLDL treatment when compared to LDL group where the LDL group displayed decreased expression compared to control; however, the alterations in αSMA were statistically not significant for both the groups (Figures 3A and 3D).

The protein expression of IL18 was significantly increased in oxLDL treatment compared to control whereas the increase in IL18 expression exhibited by LDL group was statistically not significant. In addition, the oxLDL group displayed significantly increased level of IL18 than LDL group (Figures 3A and 3D). The expression of S100B was decreased in LDL and oxLDL groups where the decrease was prominent in oxLDL treatment; however, this was statistically not significant for both the groups (Figures 3A and 3D). LOX1, TLR4 and NLRP3 displayed increased expression in LDL and oxLDL groups where the increase was found in the oxLDL treatment; however, was statistically not significant for both the groups for the three proteins (Figures 3A, 3B and 3D). Podoplanin exhibited increased expression in LDL and oxLDL groups where the increase was predominantly in the LDL treatment; however, the increase was statistically not significant (Figures 3B and 3D). Myf5 showed significantly decreased expression in both LDL and oxLDL groups where the decrease was predominantly in the oxLDL treated group (Figures 3A and 3D). Additionally, the radar plot demonstrated that the extent of the expression of the protein mediators mostly favored oxLDL treatment (Figure 3C).

### 3.3 Lipid peroxidation

**Figure 4:**
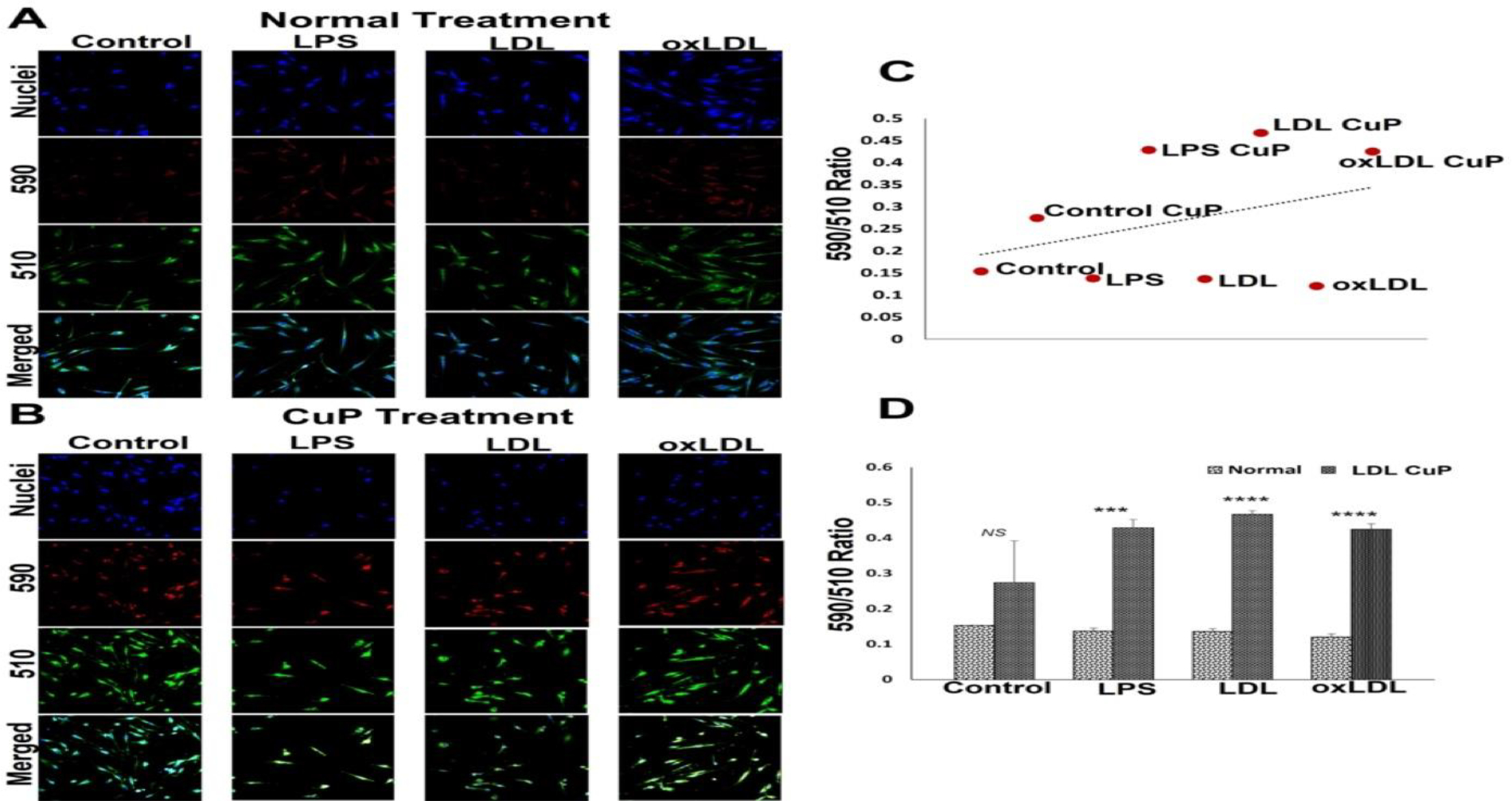
Representative images for the lipid peroxidation staining: (A) carotid SMCs treated with LPS, LDL and oxLDL and (B) carotid SMCs treated with LPS, LDL and oxLDL along with CuP. Images in the left panel represent the control group, second panel represents LPS group, third panel represent the LDL treatment group and at the right panel represent oxLDL treatment group. Images in the top row of each panel show nuclear staining with DAPI; the images in the middle rows show intensities at 590 and 510 and the lower row shows overlay staining with DAPI. Images were acquired at 20x magnification. (C) Scatter plot showing the trend of decreased lipid peroxidation in CuP treatment group. (D) The quantification of lipid peroxidation based on 590/510 ratio where the graph represents mean values with standard error. The statistical significance based on one-way ANOVA test is represented in the figure (*** *P<0.001,* **** *P<0.0001, and NS - non-significant*).

The ratiometric quantification of lipid peroxidation has been represented by the ratio of 590/510 fluorescence intensities. The extent of lipid peroxidation was similar in the LDL, oxLDL and LPS treatment groups compared to the control (Figures 4A and 4D). Surprisingly, the CuP treatment groups displayed drastic decrease in lipid peroxidation as evident from the increased 590/510; however, the increase was statistically not significant in the control group (Figures 4B and 4D). Additionally, the trend of lipid peroxidation was lower in normal treatment group when compared with CuP treatment (Figure 4C) suggesting the potential of SMCs to withstand the pro-lipid peroxidation reactions.

### 3.4 Mitochondrial membrane integrity

**Figure 5:**
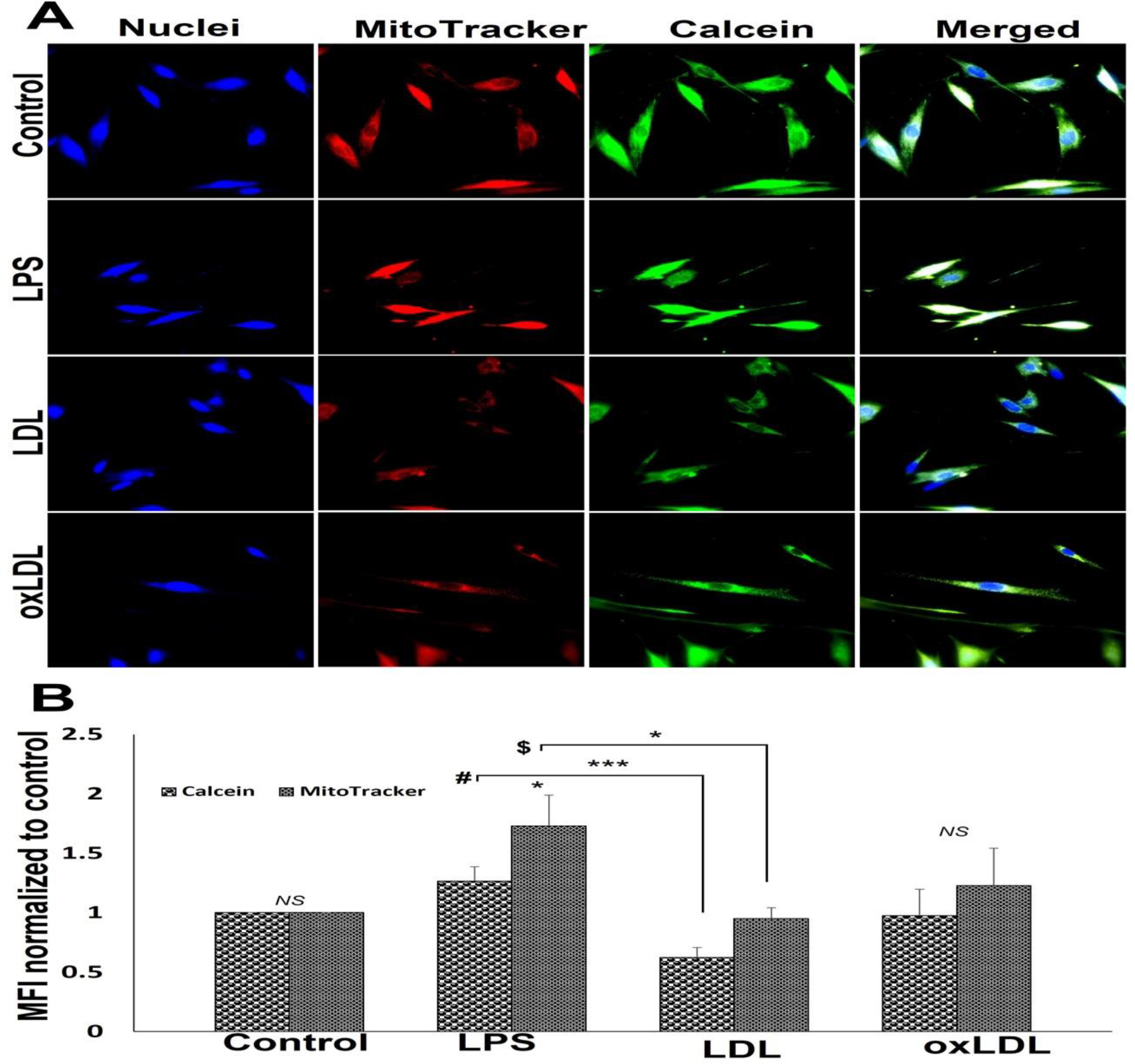
Representative images for the mitochondrial pore transition staining: (A) control SMCs and carotid SMCs treated with LPS, LDL and oxLDL. Images in the left panel represent the nuclei, second panel represents MitoTracker red staining, third panel represents calcein staining and the right panel shows overlay staining with DAPI. Images in the top row of each panel show control cells, second row displays LPS group, third row represents LDL treatment, and the fourth row shows oxLDL treatment. The images were acquired at 40x magnification. (B) The quantification of MitoTracker red and calcein staining where the graph represents mean values with standard error. The statistical significance based on one-way ANOVA test is represented in the figure (*** *P<0.001,* * *P<0.05,* # *LPS vs LDL for calcein, $ LPS vs LDL for MitoTracker and NS - non-significant*).

Treatment with LDL and oxLDL imparted alterations in the mitochondrial membrane integrity of SMCs. The MFI corresponding with calcein intensity was higher in the LPS treatment group, decreased in LDL group, and oxLDL group displayed similar level of MFI compared to control; however, alterations in calcein intensity was statistically not significant (Figures 5A and 5B). Moreover, LDL group displayed a significant decrease in calcein intensity compared to LPS group (Figures 5A and 5B). Since the healthy mitochondria are impermeable to CoCl_2_ present in the reagent mix, which is capable of quenching calcein fluorescence, the decreased MFI for calcein in the LDL-treated SMCs signifies the compromised mitochondrial membrane integrity. The intensity of MitoTracker fluorescence was increased in LPS and oxLDL groups whereas the increase in oxLDL group was statistically not significant when compared to the control suggesting an increased mitochondrial density (Figures 5A and 5B). Additionally, MitoTracker intensity in LDL group was like the control (Figures 5A and 5B).

### 3.5 Microtubule and cell spreading assay

**Figure 6:**
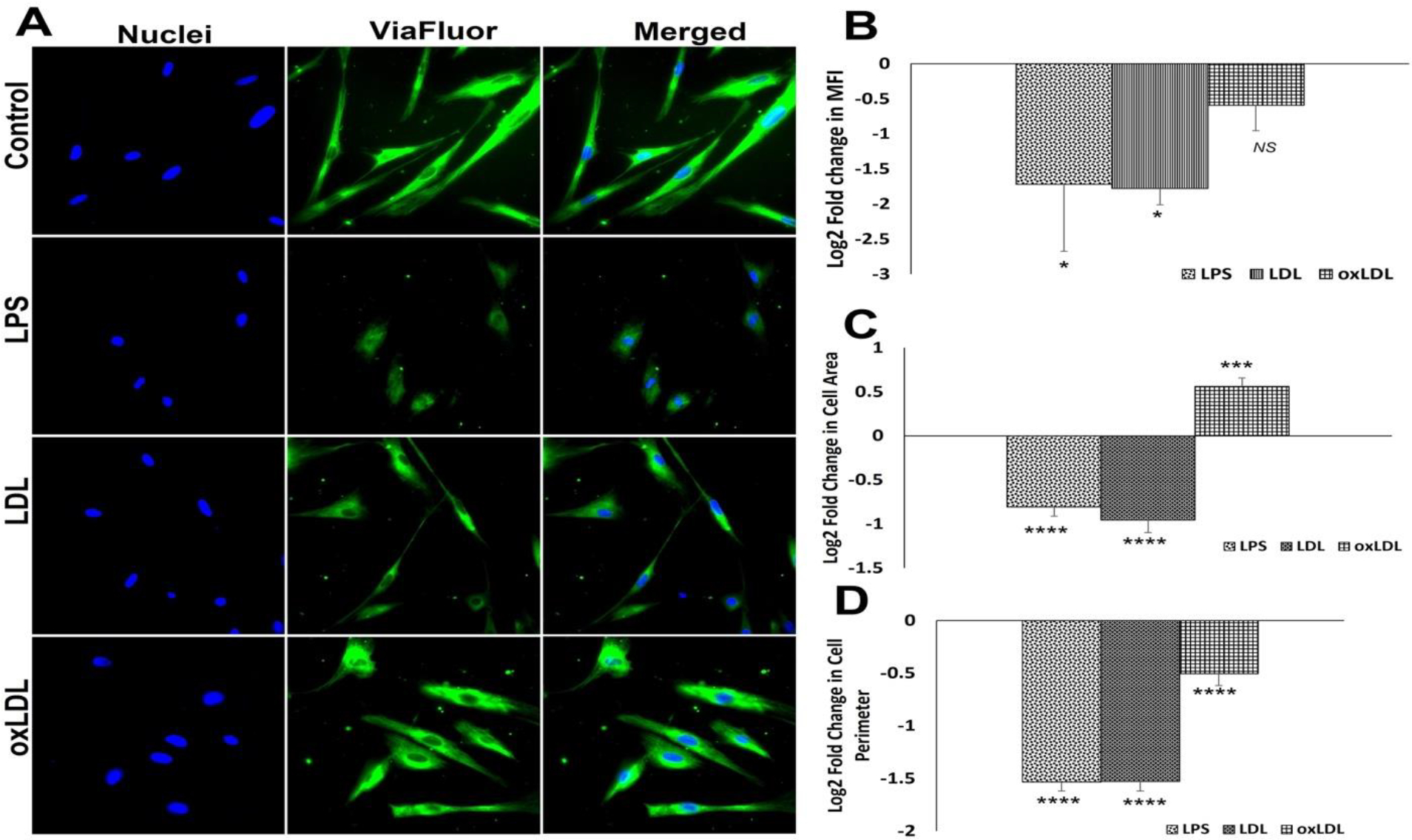
Microtubule staining by Viafluor: (**A**) Representative images for Viafluor staining showing control SMCs and carotid SMCs treated with LPS, LDL and oxLDL. Images in the left panel represent the nuclei, middle panel represents Viafluor staining, and the right panel shows overlay staining with DAPI. (**B**) The quantification of Viafluor staining where the graph represents mean values with standard error. The quantification of area (**C**) and perimeter (**D**) from the Viafluor stained images. The statistical significance based on one-way ANOVA test is represented in the figure (**** *P<0.0001,* *** *P<0.001,* * *P<0.05, and NS -* ).

A significant drop in the expression of microtubules was observed in the LPS- and LDL-treated SMCs compared to the control as evident from the decreased Log_2_ FC in MFI. However, the decreased MFI in oxLDL treatment group was not statistically significant. In addition, the oxLDL group displayed increased MFI compared to LPS and LDL groups; however, was not statistically significant (Figures 6A and 6B). LPS- and LDL-treated SMCs displayed a significant decrease in cellular area compared to the control whereas oxLDL treatment displayed a significant increase in cellular area (Figures 6A and 6C). Interestingly, the cellular perimeter was significantly lower in LPS-, LDL- and oxLDL-treated groups than the control group. Additionally, oxLDL group displayed significant increase in perimeter compared to LPS and LDL groups (Figures 6A and 6C). Overall, the data suggest that LDL induces more impact on microtubule biology when compared to oxLDL.

## 4. Discussion

Similar to coronary arteries, the carotid arteries are highly affected with atherosclerosis displaying similar pathological features irrespective of the anatomical and hemodynamic differences [25]. Carotid stenosis due to atherosclerosis and the plaque instability, especially at the bifurcation (carotid-junction), have been identified to be the major risk factor for cerebrovascular diseases including stroke owing to the carotid plaque rupture [26]. Importantly, the similarities in the anatomy, physiology and pathological response between swine and human heart/cardiovascular system and the relatively larger size make swine model to be an ideal choice for atherosclerosis. In addition, the swine model permits the use of same surgical tools, interventional approaches, and management strategies as in human patients and the swine atherosclerosis pathology closely simulates clinical atherosclerosis. Hence, the present study utilized Yucatan microswine [27, 28].

Pathologically, oxLDL aggravates carotid atherosclerosis by accelerating pro-inflammatory events and foam cell formation by macrophages and SMCs [29, 30]. The minimally oxLDL has been prevalent in the plaque and circulation than the fully oxLDL suggesting their pathological significance. Interestingly, minimally oxLDL are not recognized by scavenger receptors for the downstream pathological signaling [31]. Owing to the pathological significance, the present study was designed using minimally oxLDL focusing on the atheroma at carotid bifurcation in clinically relevant hyperlipidemic Yucatan microswine model of carotid atherosclerosis and isolated SMCs following the standard staining protocols.

OxLDL triggers the upregulation of cell adhesion molecules and activates the endothelial cells and SMCs resulting in the recruitment, homing, and differentiation of circulatory leukocytes. In addition, the pro-inflammatory milieu created by hyperlipidemia triggers further oxidation of LDL leading to lipid burden in the intima. Consequently, the activation of macrophages results in the increased pool of pro-inflammatory cytokines, reactive oxygen species (ROS), and proteolytic and/or oxidative enzymes which promote the disorganization of vascular ECM. Subsequently, neointimal hyperplasia (NIH) and necrotic core formation occur which matures to form plaques in the carotid arteries [32]. Interestingly, the histological investigations revealed fibro-adipo-necrotic core formation in the carotid arteries treated with oxLDL suggesting the translational significance of our animal model. Moreover, the histological findings closely simulated the typical clinical presentations observed in human carotid atherosclerosis [33, 34].

Importantly, the cellular necrosis owing to the hostile biochemical niche resulting from the lipid burden drives the release of DAMPs aggravating the atheromatous reactions via the signaling through the mediators including IL18, S100B, PDGFRα, HMGB1, IL17, LOX, TLR4, Myf5, IL-1β, and podoplanin (7–16). Interestingly, the expression status of these mediators was altered in the carotid arteries of oxLDL-treated group compared to the control. Also, the major DAMPs including HMGB1 and S100B and their downstream signaling via TLR4 receptor have been intimately associated with the aggravated atherosclerosis resulting in the upregulation of proinflammatory cytokines including IL-18 and IL-1β [35, 36].

Surprisingly, the carotid artery specimen revealed a decreased expression of HMGB1 and S100B with a concomitant increase in the pro-inflammatory cytokines IL-17, IL-18 and IL-1β suggesting the priming role of DAMPs in initiating carotid atherosclerosis. Moreover, extracellular proteases degrade these DAMP proteins and resolution of inflammation and/or necrosis minimize their local release as the tissue harvest occurred >4 months following the initial injury. Interestingly, the treatment of oxLDL upregulated HMGB1 in cultured SMCs unveiling the DAMP effect; however, S100B was decreased in both LDL and oxLDL groups suggesting HMGB1 to be the strong mediator in triggering sterile inflammation via the activation of NLRP3 inflammasome in carotid atherosclerosis. Hence, it is reasonable to hypothesize that the necrotic cells release DAMPs which facilitate TLR4 signaling to sustain the activation and translocation of NF-κB resulting in the upregulation of a battery of pro-inflammatory cytokines. Among the cytokines IL-17, IL-18 and IL-1β, IL-18 has been reported to be potent in inducing and sustaining inflammatory diseases including atherosclerosis [37, 38]. Interestingly, on encountering oxLDL, the cultured SMCs displayed increased level of IL-18 than IL-1β supporting the superior pro-atheromatous potential of IL-18.

PDGF signaling is a critical risk factor for NIH which triggers the activation, proliferation, and migration of SMCs via PDGFRα receptor [39, 40]. Moreover, a seminal article recently reported the intimate involvement of PDGFRα-positive SMCs in the pathology of atherosclerosis by promoting NIH [41]. Importantly, the increased hemodynamic stress and the inflammatory episodes following the LDL burden activates PDGFRα further facilitating NIH [42]. The increased level of PDGFRα in oxLDL-treated carotid vessels indicates the sustenance of SMC activation and progressive NIH during the chronic phase of atherosclerosis. Interestingly, the cultured SMCs displayed decreased level of PDGFRα suggesting that sustained exposure of oxLDL is the prerequisite for SMC activation as evident in the chronic phase of atherosclerosis.

The sustenance of proinflammatory signals including TNF*α*, IL-1 and IL-18 and proatherogenic stimuli including the burden of modified lipoproteins induce the expression of LOX1. Additionally, the upregulation of LOX1 in SMCs indicates accelerated foam cell formation and intimal pathology [43]. Interestingly, LOX1 aggravates the pathology of atherosclerosis by promoting arterial thrombus formation apart from the lipid burden where the thrombosis has been a critical risk factor in cerebrovascular and cardiovascular diseases [44]. Furthermore, the high calorie diet-induced intimal pathology activates LOX1 expression which is aggravated by increased pool of oxLDL [45] supporting the translational significance of our experimental model as evident from the exasperated foam cell formation and increased level of LOX1 in oxLDL-treated carotid arteries. As expected, the cultured SMCs displayed increased LOX1 level suggesting their closer association with carotid atherosclerosis.

Myf5 expression is indicative of proliferating myoblasts/satellite cells/smooth muscle precursor cells representing actively proliferating SMCs destined in the development of NIH [46, 47]. Interestingly, the TLR4 signaling has been reported to be associated with Myf5 expression suggesting the association between pro-inflammatory milieu following the hyperlipidemia and/or intimal lipid burden with subsequent mobilization/activation of SMCs via Myf5 [48]. Similarly, the expression of podoplanin has been associated with SMCs in advanced atherosclerotic lesions (minimally in early lesions) and thrombus formation [49, 13]. Our data revealed increased expression of Myf5 and podoplanin with a concomitant increased level of TLR4 in the oxLDL-treated carotid vessels correlating with the advanced atherosclerotic lesion and the sustained progression of NIH. However, oxLDL-treated SMCs in culture displayed decreased expression of Myf5 warranting further investigation to unveil the mechanistic aspects of oxLDL-driven induction of Myf5 gene.

**Figure 7:**
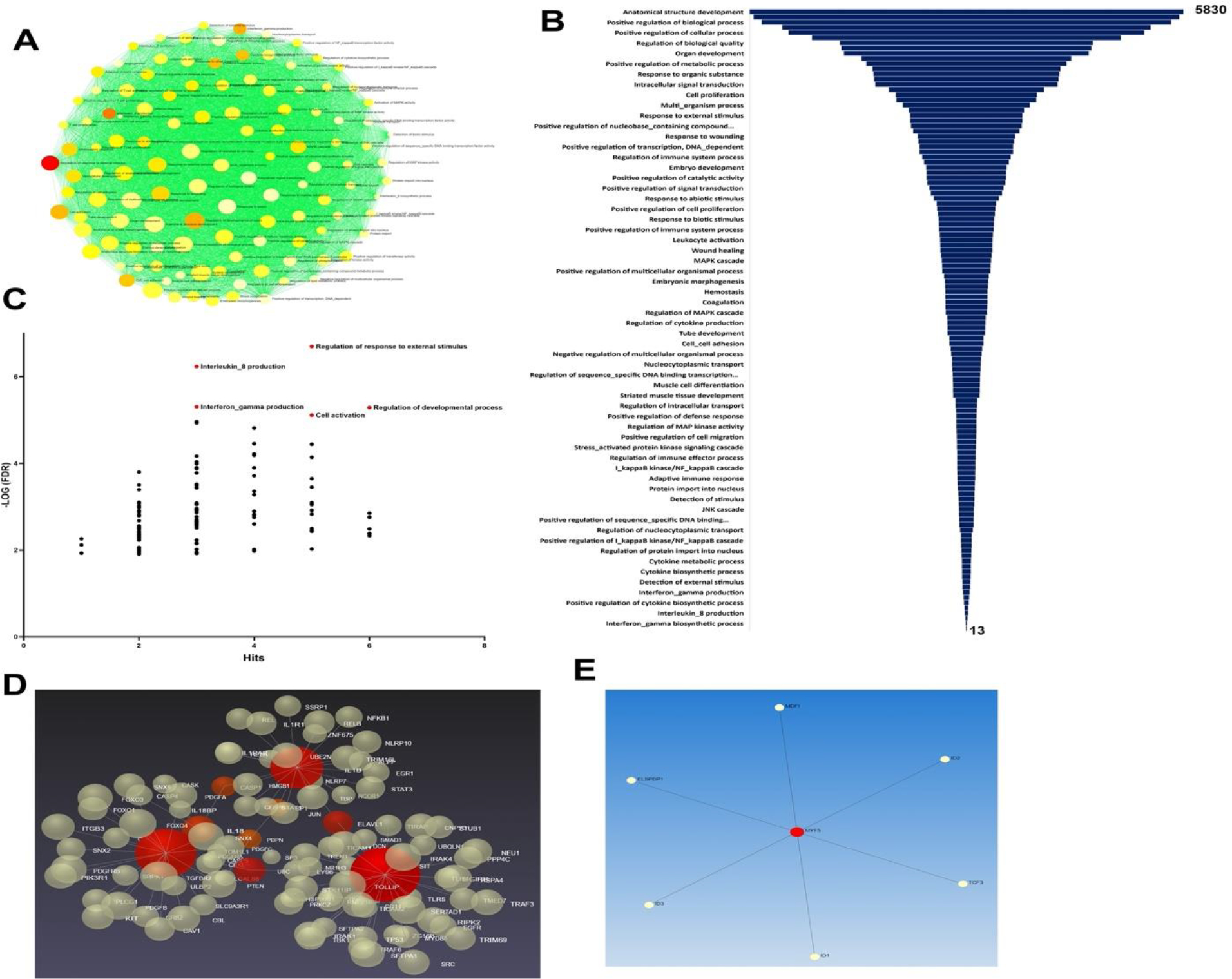
(**A**) ORA network visualization showing the interconnectivity of biological processes (GO: BP) based on the 8 input genes. (**B**) Funnel diagram displaying the total interactions for each biological process connected to the 8 input genes (*P<0.05 for all hits*). (**C**) Volcano plot showing the key biological processes based on the −Log (FDR). (**D**) 3D visualization based on the Force-directed map showing the crosstalk between 105 genes associated with the sub-network 1 associated with the 8 regenerative input genes. (**E**) 2D visualization based on the Force-directed map showing the crosstalk between 7 genes associated with the sub-network 2 associated with the 8 regenerative input genes.

The eight major genes upregulated in the carotid vessels (IL18, PDGFRA, IL17, LOX1, TLR4, MYF5, IL1B, and PDPN) were intimately involved in the pathologic progression of atherosclerosis and NIH. However, the priming signals such as HMGB1 and S100B were downregulated indicating the chronic phase of the pathology. Taken together, the decreased level of S100B in cultured SMCs suggests its minimal involvement in atherosclerosis pathology; however, further investigations are warranted. The upregulated genes were associated with 120 pathways as assessed by ORA (Over Representation Analysis) based on Gene Ontology: Biological Processes (GO:BP) (Figure 7A).

The volcano plot revealed several predominant biological events associated with the upregulated genes, including ‘Regulation of response to external stimuli’, ‘Production of IL-8 and IFN-γ’, ‘Regulation of developmental processes’, and ‘Cell activation’ (Figures 7B and 7C). The artery specific interactions revealed two sub-networks. Sub-network 1 is associated with 120 pathways and 105 genes whereas the sub-network 2 is associated with 7 genes and 93 pathways (Figures 7D and 7E).

The genes and pathways of sub-networks 1 and 2 and the interconnected genes are mostly involved in immune responses and cellular homeostasis which in turn are critical in the pathology of atherosclerosis. Importantly, the oxLDL uptake is not limited to SMCs. Endothelial cells, immune cells including macrophages and adventitial fibroblast (to a lesser extent) cells in the vessels were reported to uptake LDL and express the above-mentioned mediators [50, 51, 52]. Hence, the carotid atherosclerosis is the cumulative effect of lipid burden and downstream signaling in these cells.

OxLDL is a well-known pro-oxidant and the active involvement of oxidative stress in atherosclerosis has been vividly established (53). Moreover, the ROS and oxidative stress are the major contributors for the phenotypic switch of contractile SMCs to the proliferative and metabolically active synthetic phenotype (54) (55); synthetic SMCs have been expected to be resistant to oxidative damage. As expected, the mitochondrial density owing to the decreased MitoTracker and membrane integrity (as evident from decreased calcein fluorescence due to the increased permeability of CoCl_2_) (24) were compromised in cultured SMCs and were prominent in LDL group than oxLDL treatment. Surprisingly, our data unveiled decreased lipid peroxidation in cultured SMCs in the presence of external pro-oxidant molecule suggesting the potential of SMCs in maintaining the membrane integrity under various pathological stresses; however, the underlying mechanism requires further research.

The pathological triggers including the LDL burden impart alterations in cellular morphology and volume (56) (57). Also, the SMC hypertrophy regulated by TGFβ signaling has been intimately involved in atherosclerosis pathology (58) (59). Importantly, the stabilization and depolarization of microtubules has been reported to be concomitant with cellular hypertrophy; however, the hypertrophic pathways in SMCs and their implications in atherosclerosis are largely unknown. Our data revealed that the treatment with oxLDL significantly increased the area of cultured SMCs whereas LDL treatment exhibited paradoxical effect which could be attributed to the increased level of microtubules in oxLDL-treated SMCs. These findings correlate the possible association of microtubule homeostasis in maintaining cellular area/volume on encountering pathological triggers such as lipid burden associated with atherosclerosis; however, the data regarding the molecular implications are unavailable which warrants further investigations.

Overall, the findings from this study successfully simulated the oxLDL-driven atherosclerotic pathology in minimally invasive clinically relevant Yucatan microswine by combining hyperlipidemia, endothelial injury, and local replenishment of oxLDL. Also, the study utilized immunostaining approaches to associate multiple pathological implications of oxLDL in aggravated atherosclerosis of the carotid artery.

Our study has several challenges and limitations; (i) the local bioavailability of oxLDL was not scalable owing to the scarcity of specific *in vivo* detection techniques, (ii) the implications of active mediators contained in oxLDL preparations in atherosclerosis pathology are currently unknown, (iii) albumin contamination owing to the cholesterol binding effects hurdled oxLDL preparation, and (iv) the relative small size of target vessel challenged (60). Additionally, the comparison was based on the vessels without any intervention from atherosclerotic pigs where the proinflammatory milieu was proven to be upregulated. Moreover, the cultured SMCs were exposed to oxLDL/LDL overnight whereas the tissues were harvested 4–6 months post treatment suggesting the need of increased exposure time in culture which is practically not possible owing to the solubility issues of LDL/oxLDL and the risk of cell death. Hence, the lack of normal controls and small sample size hurdled to generate statistically significant data warranting further detailed investigations. Nonetheless, the current study established a strong association of oxLDL in driving diverse biological processes in the pathology of atherosclerosis in a translationally relevant pre-clinical model and further detailed investigations would open novel opportunities in atherosclerosis management.

## 5. Conclusions

**Figure 8:**
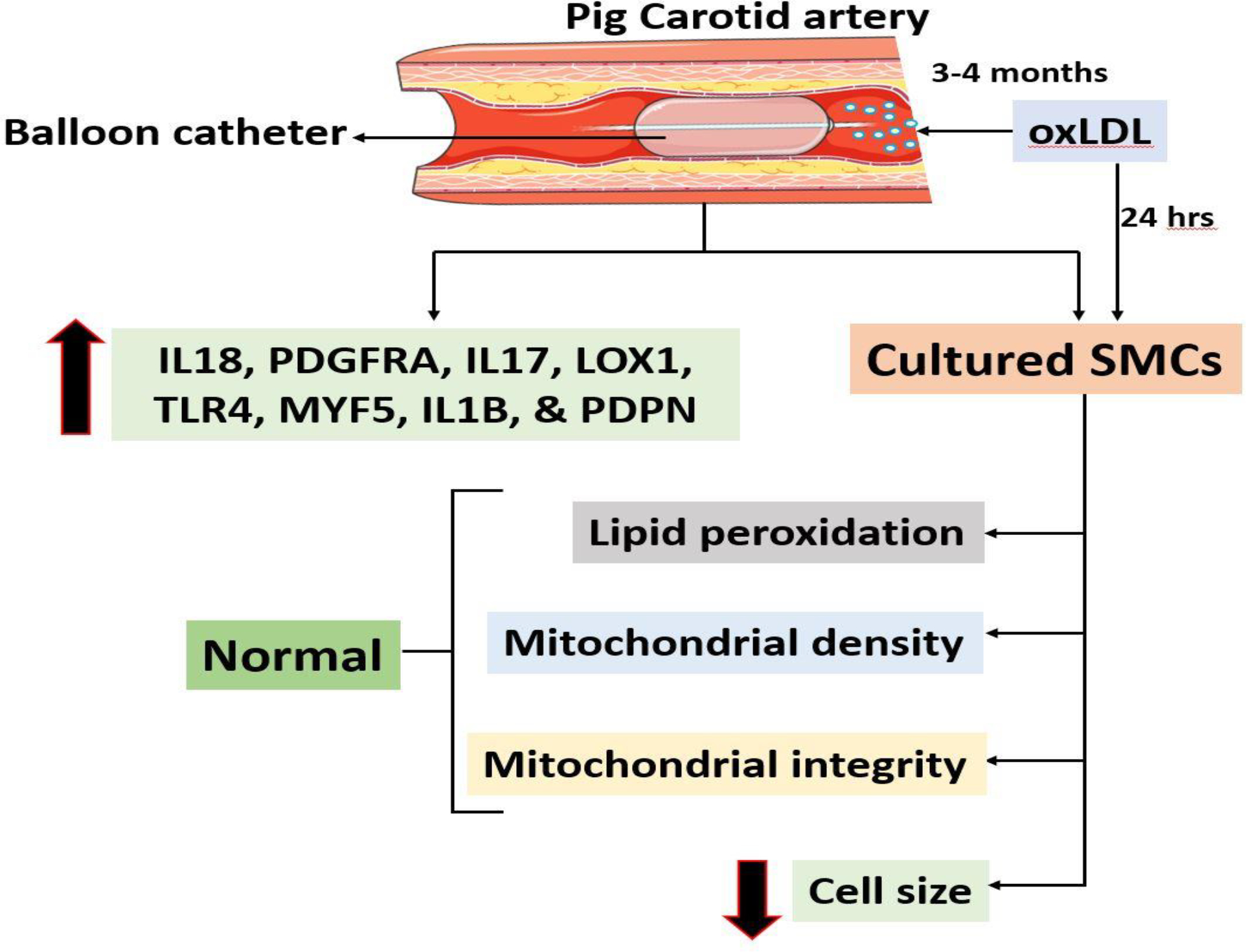
The schematic representation of overall findings from the study.

The pro-atherogenic effects of oxLDL was successfully replicated in the carotid arteries in our translationally relevant minimally invasive hyperlipidemic Yucatan microswine model and the vascular alterations were examined using multiple staining approaches. The expression of pro-inflammatory and pro-atheromatous mediators including IL18, PDGFRA, IL17, LOX1, TLR4, MYF5, IL1B, and PDPN were increased in the carotid artery bifurcation. However, the level of priming DAMPs, HMGB1 and S100B was decreased in the carotid vessels indicating the chronic phase of the pathology. The cultured SMCs displayed increased expression of IL18, LOX1, TLR4, MYF5, NLRP3, and PDPN on encountering oxLDL. In addition, the SMCs treated with oxLDL were resistant to the peroxidation of lipids; however, displayed compromised mitochondrial membrane potential and increased hypertrophy due to decreased level of microtubules. The overall findings are depicted in Figure 8. Overall, the oxLDL exerts multiple biochemical events in carotid SMCs signaling the atherosclerosis pathology and the understanding regarding the molecular mechanisms underlying such pathological events would open novel translational avenues in the management of carotid atherosclerosis.

## Data Availability

Data with the raw counts matrices and annotation are available upon request from the authors through proper channels.
